# MicroRNA* ame-let-7* targets *Amdop2* to increase sucrose sensitivity in honey bees (*Apis mellifera*)

**DOI:** 10.1186/s12983-023-00519-7

**Published:** 2023-12-18

**Authors:** Fang Liu, Hongxia Zhao, Qiang Li, Lixian Wu, Dainan Cao, Yuan Zhang, Zachary Y. Huang

**Affiliations:** 1 Guangdong Key Laboratory of Animal Conservation and Resource Utilization, Guangdong Public Laboratory of Wild Animal Conservation and Utilization, Institute of Zoology, Guangdong Academy of Sciences, Guangzhou, 510260 People’s Republic of China; 2 Yunnan Academy of Biodiversity, Southwest Forestry University, 650224 Kunming, Yunnan People’s Republic of China; 3 Department of Entomology, Michigan State University, East Lansing, MI 48824 USA

**Keywords:** Honey bee, *Amdop2*, *Ame-let-7*, *Apis mellifera*, Sucrose responsiveness

## Abstract

**Background:**

As an important catecholamine neurotransmitter in invertebrates and vertebrates, dopamine plays multiple roles in the life of the honey bee. Dopamine receptors (DA), which specifically bind to dopamine to activate downstream cascades, have been reported to be involved in honey bee reproduction, division of labour, as well as learning and motor behaviour. However, how dopamine receptors regulate honey bee behavior remains uninvestigated.

**Results:**

The expression level of *Amdop*2 in the brain increased with the age of worker bees, which was just the opposite trend of *ame-let-7*. Inhibition of *ame-let-7* through feeding an inhibitor upregulated *Amdop2* expression; conversely, overexpression of *ame-let-7* through a mimic downregulated *Amdop2*. Moreover, knockdown of *Amdop2* in forager brain led to significantly higher sucrose responsiveness, which is similar to the phenotype of overexpression of *ame-let-7*. Finally, we confirmed that *ame-let-7* directly targets *Amdop2 *in vitro by a luciferase reporter assay*.*

**Conclusions:**

*ame-let-7* is involved in the dopamine receptor signaling pathway to modulate the sucrose sensitivity in honey bees. Specifically, it down-regulates *Amdop2*, which then induces higher responses to sucrose. These results further unraveled the diverse mechanisms of the dopamine pathway in the regulation of insect behavior.

**Supplementary Information:**

The online version contains supplementary material available at 10.1186/s12983-023-00519-7.

## Background

Dopamine (DA) is an important neurotransmitter that has been strongly implicated in the regulation of locomotor activity, sexual behaviour, development and endocrine function in vertebrates and invertebrates [1]. DA interacts with dopamine receptors, enabling downstream chemical responses. Vertebrates have five subtypes of dopamine receptors: D1-like (D1 and D5) and D2-like (D2, D3 and D4) receptors [2], which have been extensively studied in mammalian brain and spinal cord [1, 2]. There are four subtypes of dopamine receptors in insects: the D1-like dopamine receptor (Dop1), the invertebrate-type dopamine receptors (Dop2), the D2-like dopamine receptor (Dop3) and the DopEcR [3]. Dopamine receptor expressed in mushroom bodies in the fly and Dop1 in the cricket are involved in olfactory learning and memory [4, 5]. DA-Dop1 signalling in the *Locusta* brain induces gregariousness, whereas DA-Dop2 signalling induces solitariness [6]. In the honey bee (*Apis mellifera*), DA is associated with reproduction, division of labour, learning, circadian rhythms and sex-specific behaviours [7]. Recent research has reported that DA can induce food craving in the honey bee similar to humans [8].

There are two known types of DA receptors in the honey bee, D1-like receptors, which includes AmDop1, and D2-like receptor AmDop3. *Amdop1* and *Amdop2* code for G-protein-coupled receptors that, when activated cause increased intracellular levels of cAMP, whereas *Amdop3* receptors cause a decrease in cAMP [9, 10]. The expression levels of these three receptors change significantly with age and caste in the brain of bees. *Amdop1* has especially low expression levels in 15-day-old bees, *Amdop2* levels in the antenna were variable, especially during the first week of adulthood [11]. It is speculated that *Amdop3* may curtail the activation of the ovary directly or indirectly through a QMP component homovanillyl alcohol [12]. *Amdop3* receptors can be activated by the queen mandibular pheromone (QMP), resulting in blockade of aversive learning of young worker bees [9]. The application of vertebrate D1-like and D2-like receptor blockers in worker bees suggests that dopaminergic receptors could decrease aversive learning in bees [13]. The putative dopamine/ecdysone receptor, *Amgpcr19*, has high expression levels in seminal vesicles suggesting a possible function in sperm transfer and storage in drones [14, 15]. Knockdown of *Amdop2* through injection of dsRNA into the mushroom bodies causes honey bees to spend less time walking but does not affect flying, fanning and upside-down behaviours [16]. Collectively, the functions of DA receptors in honey bees are well studied, but the mechanisms by which DA receptors modulate honey bee behaviors remain unknown.

MicroRNAs (miRNAs) are small (18~24-nucleotide) noncoding, single stranded RNA, which can regulate gene expression by binding complementarily with target mRNA [17]. They play important roles in almost all biological process in eukaryotes [18, 19]. DA-receptors being regulated by miRNAs have been well studied in mammals. For instance, *miR-9* regulates the dopamine receptor D2 expression to enhance stress susceptibility and resistance to escitalopram treatment in rats [20]. Overexpression of miR-124 promotes dopamine receptors D1 and D2 and neuronal proliferation and suppresses neuronal apoptosis in rats [21]. *MiR-217* activates the dopamine D2 receptor to protect fibrosis in human renal proximal tubule cells [22]. One study suggested that miRNA targets the dopamine receptor involved in progression of endometrial cancer [23]. In contrast to mammals, few studies were conducted on dopamine receptors in insects. Guo et al. (2018) reported that *Dop1* inhibited *miR-9a* to modulate locust olfactory attraction by inducing the dissociation of La protein [24]. However, there was no report about the interaction between dopamine receptor and miRNA in the honey bee.

Proboscis extension response (PER) is a behavior of a honey bee responding by extending her proboscis when a drop of sugar solution is applied to her antennae [25]. The response of honey bees to different concentrations of sucrose can be tested by the PER assay. Responsiveness to sucrose is associated with foraging and collecting-choices. Nurses show weak response to sucrose, while pollen foragers show stronger response to sugar than nectar foragers [26, 27]. In a previous study, nurse brain was shown to have higher expression of *ame-let-7* than foragers [28]. Bioinformatic analyses suggested that *Amdop2* was the target of *ame-let-7*. We therefore hypothesized that *ame-let-7* regulates *Amdop2* which in turn regulates sucrose response in honey bee workers. Because PER to sucrose is higher in foragers than nurses, we also hypothesized that *Amdop2* might correlate with behavioral development in honey bees. Specifically *Amdop2* should be high in foragers and it should also enhance PER in honey bees.

## Materials and methods

### Honey bee sample collections

Three European honey bee (*Apis mellifera*) colonies were maintained according to standard beekeeping practices at the Institute of Zoology, Guangdong Academy of Sciences, Guangzhou, China (23.9325°N, 113.2935°E). One-day-old honey bees were obtained by removing a frame of capped pupae from a typical colony to an incubator (34 °C) until adults emerged. Each one-day-old honey bee was painted with a bee-marking pen and kept in the incubator for an hour before being put back into the original colony. A total of 1000–1500 one-day-old honey bees were marked from each colony. Fifteen worker bees were collected at ages 1, 6, 11, 14, 21, 25 and 30 d (with the day of emergence as day 1), and their brains were dissected immediately and stored at − 80 °C for total RNA extraction. Bees 14 days and older were collected as foragers. Foragers were identified as returning bees with pollen on their corbiculae and captured at the entrance.

### Oversupply/inhibition of ame-let-7 in honey bees

A mimic of *ame-let-7* with the sense strand (5′ ugagguaguagguuguauagu3′) and the antisense strand (5′ uauacaaccuacuaccucauu3′) including a 2nt-3′ overhang (UU) and 2 nt-5′trim was synthesized by GenPharma (Shanghai, China). An inhibitor (5′ acuauacaaccuacuaccuca3′), a single stranded RNA exactly complementary to *ame-let-7* sequence was also synthesized. A mimic control by using nonsense sequence (sense: 5′ uucuccgaacgugucacgutt 3′; antisense: 5′ acgugacacguucggagaatt 3′) and an inhibitor control using nonsense sequence (5′ caguacuuuuguguaguacaa 3′).

To overexpress or inhibit the expression of *ame-let-7* in honey bees, 30 foragers (25-day-old) were fed with 3.3 μg mimic of *ame-let-7* in 10 μl 50% sucrose solution, another 30 bees were fed with 3.3 μg *ame-let-7* inhibitor. The same amount of mimic control sequence (n = 30) or inhibitor control sequence (n = 30) was also fed to foragers as controls. Thus there were four groups of bees, fed either with *ame-let-7* mimic (*let-7M*), or its inhibitor (*let-7I*), nonsense sequences of mimic (*let-7M-NS*) and nonsense sequences of inhibitor (*let-7I-NS*). Foragers were cold-anaesthetized, individually secured in 0.5-ml Eppendorf tubes with a strip of electric tape, and kept in an incubator (28 °C, 70% relative humidity) for at least an hour to recover. The feeding treatments were repeated with foragers from three different colonies. All the foragers were fed to satiety with 50% sucrose solution 3 h after treatments, and kept in an incubator in darkness (28 °C, RH 70%). After 24 h, foragers were tested for sucrose responsiveness using the proboscis extension reflex (PER) assay [30]. Both antennae of foragers were touched with a droplet of ascending concentrations of sucrose: 0.1, 0.3, 1, 3, 10 and 30% (w:w) to test their sucrose responsiveness. Bee brains were dissected immediately after PER for total RNA extraction.

### RT-PCR and qRT-PCR analyses

Total RNA was extracted using Trizol (Invitrogen) protocol. The quality and quantity of RNA were determined using a NanoDrop (Thermo Fisher Scientific, Wilmington, DE, USA), before being stored at − 80 °C. Total RNA (1 μg per sample) was reverse-transcribed with mRQ Buffer (2 ×) and mRQ enzyme according to the Mir-X miRNA first-strand synthesis kit (Takara, Japan). The qPCR (quantitative polymerase chain reaction) assays were performed on an ABI StepOnePlus™ Real-Time PCR system. Amplification was carried out in 20 μl reaction volumes, containing 10 μl TB Green Premix Ex Taq II (2 ×), 0.4 μl forward primer (10 μM), 0.4 μl reverse primer (10 μM), 6.2 μl ddH_2_O, and 3 μl cDNA (0.5 μg). Reaction conditions were 95ºC for 30 s, followed by 40 cycles of 95ºC for 5 s and 60ºC for 30 s, followed by a melting curve (55–95 °C). *β-actin* and *GADPH were* used as reference genes for *Amdop2*, and a small RNA *u6* was used as reference gene for *ame-let-7*. For each gene, test reactions were performed in triplicates. Relative gene expression was calculated using the 2^−△△Ct^ method [31].

### RNA interference

To knockdown *Amdop2* expression, double stranded RNA (dsRNA) was synthesized using T7-RiboMAXTM Express RNAi System (Promega, USA) according to the manufacturer’s instructions. Thirty foragers were each fed with 10 μl 50% sucrose solution containing 2 μg dsRNA. Another 30 foragers were each fed with the same amount of ds*GFP* as a control. After 24 h, foragers were tested for sucrose responsiveness using PER assay. Bee brains were dissected immediately after PER. The dissection was done in cold saline following that of Olivier et al. [32]. These brains then immediately extracted for total RNA using the Trizol method. The expression of *Amdop2* was analyzed by qPCR as described above. The primers for RNAi were listed in Table 1.

**Table 1:** Primers used in this study for *Amdop2* double-stranded RNA synthesis, reverse-transcription quantitative polymerase chain reaction (RT-qPCR) analyses and construction of luciferase reporter vector

Gene	Application	Primer sequence (5′–3′)	Amplicon size (bp)	TM (°C)
*Amdop2*	RNA interference	F: CAGGCCTGGCTATACTCCTG R: GTCGGTGATGGCCCAGTA	314	55
		T7F: CAGGCCTGGCTATACTCCTG T7R: GTCGGTGATGGCCCAGTA	350	58
*Amdop2*	qPCR	F: CAAGACGTTGGGGATCGTGA R: GATCCAACCCAGCCACGTAA	142	55
*β-actin*		F: TGCCAACACTGTCCTTTCTG R: AGAATTGACCCACCAATCCA	138	55
*GAPDH*		F:CACCTTCTGCAAAATTATGGCG R:ACCTTTGCCAAGTCTAACTGTTAA	156	55
*ame-let-7*		F: GCATGTGAGGTAGTAGGTTG R: GTGCAGGGTCCGAGGT	21	55
*Amdop2-CDS*	Luciferase reporter assay	F: GAGTAAGGCGGCGGTATCAA R: TTTGCTCGCACGAACTCTCT	420	59

F, forward primer; R, reverse primer

### Dual luciferase reporter assay

The *Amdop2* coding sequence fragments of 420-bp containing *ame-let-7* binding sites (Additional file 2: Fig. S1) and its mutant sequence (*Amdop2*-CR-mut) were synthesized and amplified using 2 × PCR Mix (Takara) (Additional file 1: Table S1), then integrated into a psiCHECK-2 dual-luciferase vector using XhoI and ApaI sites to form the psiCHECK-2-*Amdop2*-CR-wide-type (*Amdop2*-CR-wt) or psiCHECK-2-*Amdop2*-CR-mutant (*Amdop2*-CR-mut) reporter vector (Table 1). HEK293T Cells (Rochenpharm, China) were seeded at 1 × 10^6^ cells per well in a 12-well plate in the 37 °C incubator. According to the manufacturer’s instructions, cells were co-transfected recombinant psiCHECK-2 luciferase reporter vector with CR of *Amdop2* (wt or mut) and *ame-let-7* mimics using lipofectamine 2000 (Invitrogen, Thermo Fisher Scientific). The control group was co-transfected with 1 μg recombinant psiCHECK-2 luciferase reporter vector with CR of *Amdop2* (or mut) and negative control of mimics (*ame-let-7* mimics NS). In all cases, 60 ng miRNA and 60 ng transfer vector were mixed, and 1.5 μg of pCopia-Renilla luciferase was added as an internal control. Twenty-four hours after transfection, luciferase assays were performed using a dual-specific luciferase assay kit (#RG027, Biyuntian, Shanghai, China). Renilla luciferase activity provided normalization for firefly luciferase activity.

### Statistical analyses

Statistical analyses were conducted in SPSS 16.0 (SPSS Inc., Chicago, IL, USA). One way analysis of variance (ANOVA) followed by Turkey’s honestly significant difference (HSD) test was used to compare the gene expression of *Amdop2* among different aged bees. ANOVA was also used to analyze the data with PER response as a dependent variable, where PER response (%) was analysed after arcsine-square root transformation. Different sugar concentrations were treated as repeated measures. Student’s T-test was used to compare the differences in *Amdop2* expression between ds*Amdop2* bees and dsGFP bees. All data are presented as the means ± standard errors (SE). A *P* < 0.05 was considered statistically significant.

## Results

### Abundance of ame-let-7 and expression patterns of Amdop2 in adult bees

**Fig. 1:**
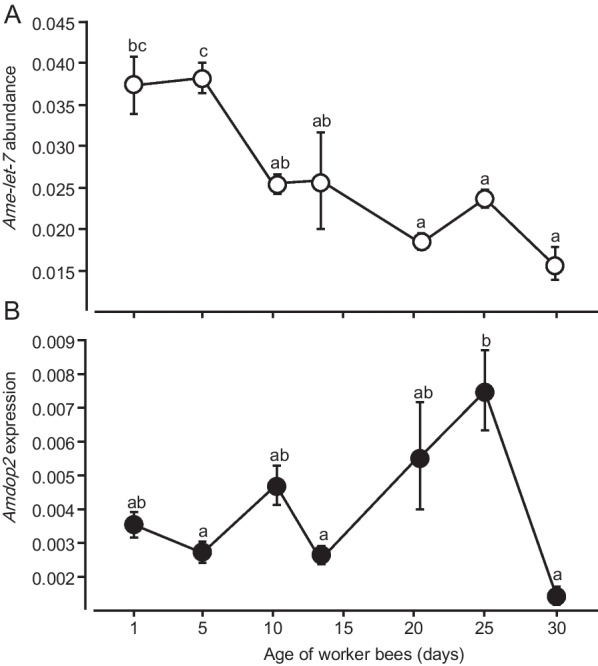
Mean (± SE) of *ame-let-7 abundance* (**A**) and expression levels of *Amdop2* (**B**) in the brains of different aged worker bees. Workers were collected at 1, 6, 11, 14, 21, 25 and 30 days post-eclosion, with those 14 days or older as foragers which returned home with pollen. Gene levels in different aged bees were analyzed by One-Way ANOVA, followed by *post-hoc* Turkey test for multiple comparisons (*n* = 3 for each point, *P* < 0.05)

*Ame-let-7* abundance was significantly different among bees of different ages (ANOVA, F = 9.18, *df* = 6,14; *P* < 0.01). In general, *ame-let-7* decreased as the age of honey bees increased, with the abundance significantly lower after day 21 compared to day 1 and day 6 (Fig. 1A). *Amdop2* expressions were significantly different among bees of different ages (ANOVA, F = 5.89, *df* = 6, 13; *P* < 0.01). In contrast to *ame-let-7*, HSD tests showed that *Amdop2* increased slowly with age of the adult bees, with a significantly higher expression at 21 and 25 days of age, but returned to the very low level at age 30 (Fig. 1B).

### *ame-let-7 regulates the expression of Amdop2 *in vivo

**Fig. 2:**
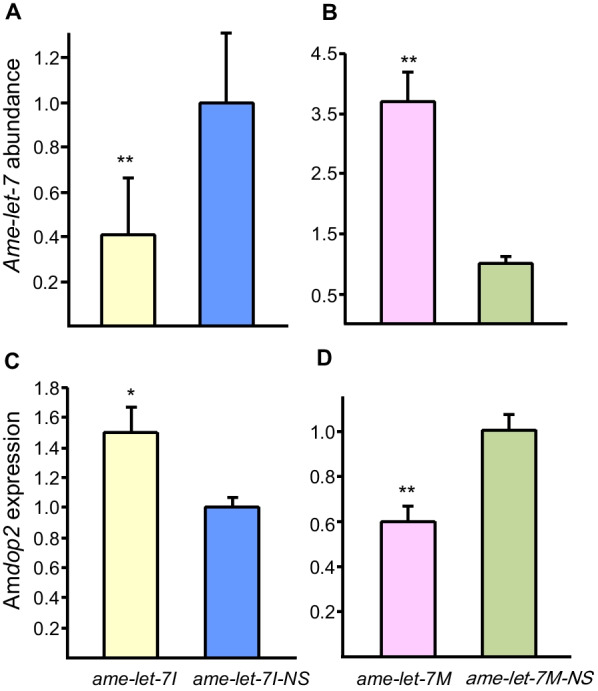
Expression levels of *ame-let-7* (**A**, **B**) and *Amdop2* (**C**, **D**) 24 h after being treated with ame-let7 mimic, mimic control, inhibitor and inhibitor control, shown as *ame-let-7 M, ame-let-7 M-NS, ame-let-7I* and *ame-let-7I-NS, respectively*. The qPCR data are presented as the mean ± SE (n = 3), ** indicates significant difference at P < 0.01, and * indicates significant difference at P < 0.01 compared with the respective NS (nonsense) groups

Brain *ame-let-7* abundance was significantly reduced in foragers after being fed with an inhibitor of *ame-let-7* (*ame-let-7I*) than its control group (*ame-let-7*I-NS) (t = 10.58, *P* < 0.01, Fig. 2A), while *Amdop2* expression was significantly enhanced in forager brains in the inhibitor-fed (*ame-let-7I*) group than the control (*ame-let-7I-*NS) group (t = 2.76, *P* < 0.05, Fig. 2C). Conversely, brain *ame-let-7* abundance was significantly enhanced when bees were fed with an *ame-let-7* mimic (*ame-let-7M)* compared to the control (*ame-let-7M-NS*) group (t = 5.92, *P* < 0.05, Fig. 2B); *Amdop2* expression showed a significant decrease in foragers, when bees were fed with a mimic of *ame-let-7* (*ame-let-7M*) compared to its control *(ame-let-7M-NS*) (t = 4.58, *p* < 0.001, Fig. 2D).

### Confirmation of the interaction of ame-let-7 with Amdop2

**Fig. 3:**
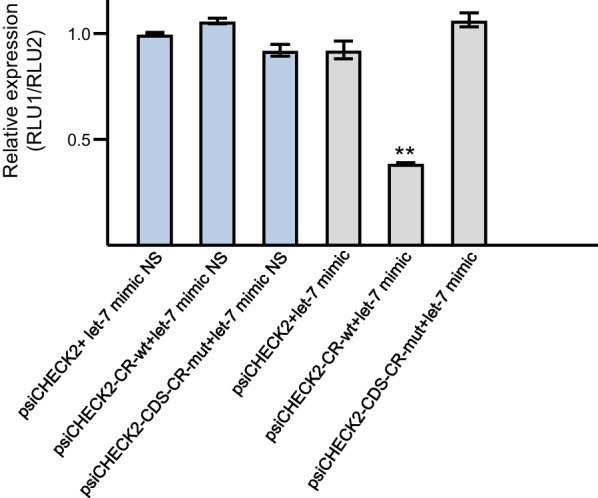
Co-transfection of psiCHECK2 *dop2-CR-wt* with *ame-let-7* mimic resulted in dramatic suppression of the luciferase activity. A normalization firefly/renilla luciferase value was plotted with ± SE (n = 3 for each point). ** indicates significant difference at P < 0.01 compared with the negative control in ANOVA analysis

When *ame-let-7* mimic was co-transfected with *dop2*-CR-wt in 293 T cells, luciferase activity was significantly decreased compared to the cells co-transfected with *dop2*-CR-m or the negative control group (F = 105.5, *df* = 5, 12; *P* < 0.0001, Fig. 3). None of the NS groups affected luciferase activity.

### ame-let-7 affects the sucrose responsiveness in foragers

**Fig. 4:**
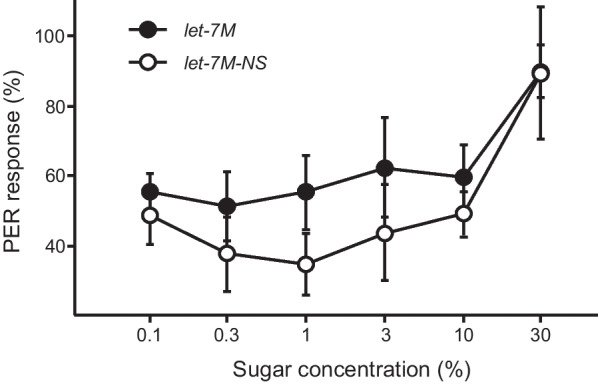
Mean score (% ± SE) of PER of bees to various sugar concentrations after being treated with a mimic of *ame-let-7* or nonsense sequences of mimic. Responsiveness to sucrose was significantly increased by a mimic of *ame-let-7*, compared with the control. Data from three colonies were analyzed after arsine-square root transformation during ANOVA but presented here without transformation. Each data point represents 30 bees

PER response varied significantly with sugar concentrations (F = 9.30, *df* = 5, 10; *P* < 0.01). PER response was significantly higher in bees fed with a mimic *(ame-let-7M*) compared to the group fed with nonsense control (*let-7*M-NS) (F = 20.4, *df* = 1, 5; *P* < 0.05, Fig. 4). There were no significant interactions between sucrose concentrations and the treatments (F = 0.52, *df* = 5, 10; *P* > 0.05).

### Amdop2 affects sucrose responsiveness in foragers

**Fig. 5:**
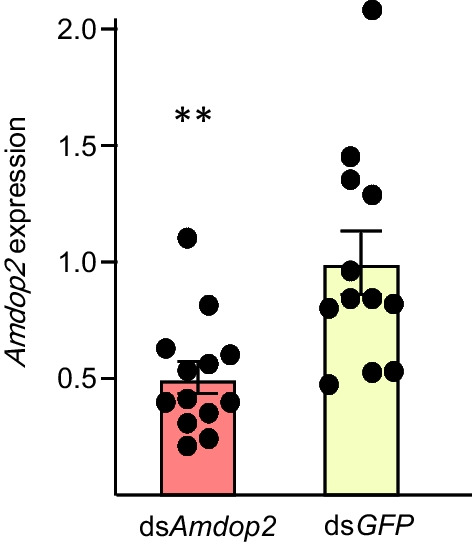
*Amdop2* expression (means ± SE) in the brains of foragers after being fed with *dsAmdop2* or a negative control ds*GFP.* ** indicates significant difference at *P* < 0.01 (Student T-test) compared with ds*GFP*

**Fig. 6:**
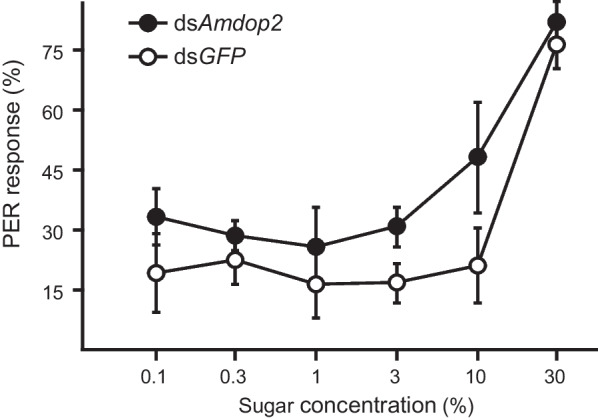
Mean score (% ± SE) of PER of bees to various sugar concentrations after being treated with ds*Amdop2* or ds*GFP* in foragers*.* Responsiveness to sucrose was significantly (*P* < 0.01) enhanced in ds*Amdop2* bees, compared to the ds*GFP* control. Data from three colonies were analyzed after arsine-square root transformation during ANOVA but presented here without transformation. Each data point represents 30 bees

The expression of *Amdop2* was significantly suppressed at 24 h (Student’s T test, t = 3.33; *P* = 0.0029) (Fig. 5), with a reduction of 50% compared to the control. PER response changed significantly with sugar concentrations (ANOVA, F = 65.07, *df* = 5, 10; *P* < 0.001) (Fig. 6). The PER response to sugar in the ds*Amdop2-*fed bees was significantly enhanced compared to the control group (F = 11.6, *df* = 1, 5; *P* < 0.001) (Fig. 6). There were no significant interactions between sucrose concentrations and the treatments (F = 1.49, *df* = 2, 5; *P* > 0.05).

## Discussion

The first major finding of this study is that *Amdop2* is regulated by *ame-let-7.* The notion that *Amdop2* was regulated by *am-let-7* was supported by several lines of evidence. First there was a reverse relationship between the two (but see below about day 30 data), with *am-let-7* decreasing with age and *Amdop2* increasing with age (Fig. 1). Furthermore, manipulating *ame-let-7* levels with a mimic (overexpression), or an inhibitor (knockdown) caused a reversed directional change in *Amdop2* expressions (Fig. 2). Lastly, we show conclusively that *Amdop2* is the target of *ame-let-7* through a luciferase assay because luciferase activity was significantly decreased when an *ame-let-7* mimic was co-transfected with *dop2*-CR-wt in HEK293T cells compared to control group (Fig. 3).

The second major finding is that *Amdop2* down-regulates sucrose responsiveness in honey bees. This was first suggested by *ame-let-7* inhibitor significantly reducing PER, presumably because in these bees *Amdop2* was increased due to a downregulation in *ame-let-7* causing an increase in *Amdop2*, which was shown earlier (Fig. 2). Conversely, an *ame-let-7* mimic significantly enhanced PER presumably because the increase of *ame-let-7* caused a decrease in *Amdop2*, which negatively affected PER (Fig. 4B). A more direct evidence was provided by the RNAi experiment where bees fed with double stranded *Amdop2* (*dsAmdop2*) showed an enhancement in PER (Fig. 6). This was after we showed that the method indeed was working, as shown by *Amdop2* being down regulated post ds*Amdop2* feeding (Fig. 5).

Our results of foragers with *Amdop2* knockdown resulting in enhanced sucrose responsiveness are consistent with other published studies. It has been shown that the injection of dopamine into the thorax significantly decreases responsiveness to sucrose in foragers [35]. In addition, thoracic injection of the dopamine receptor agonist 2-amino-6, 7-dihydroxy-1, 2, 3, 4-tetrahydronaphthalene (6, 7-ADTN) and administration of 6, 7-ADTN also significantly reduces sucrose responsiveness in foragers [33]. Up-regulation of *Amdop2* expression results in an increase in dopamine in the central brain of the honey bee, which regulates neuronal sensitivity to dopamine both temporally and spatially [34–36]. In the present study, RNAi of *Amdop2* in the brains of foragers reduced the number of dopamine receptors, causing an effect similar to decreasing dopamine titre. This decrease then made the foragers more sensitive to sugar, consistent with the increase of dopamine in reducing sucrose responsiveness [33].

It is intriguing that *Amdop2* would have a negative effect on PER, given that PER is shown to be higher in foragers than nurses [26, 27] and our first experiment here showing that *Amdop2* was higher in foraging-aged bees (21–25 days) compared to younger bees (10 days or younger, Fig. 1). It is possible that the *Amdop2* increase we observed here in Fig. 1 was not related to division of labor, but purely due to worker age. This was at least true in antennae *Amdop2* expression in nurses and foragers, which showed no differences [11]. Our own data here also suggests there was no tight link between *Amdop2* and division of labor because both 14 and 30 day old bees were collected as foragers but showed low levels of *Amdop2* (Fig. 1). However, more studies are required to confirm that changes in *Amdop2* expression is more as a function of age, rather than due to difference in behaviors.

Several miRNAs have been shown to regulate honey bee behaviors, since the first study by Weaver and colleagues [37]. *MiR-932* regulates honey bee memory by targeting *actin* [38]. *MiR-279a* regulates forager sugar responsiveness by suppressing *Mblk-1* [30, 39]. Previously, we predicted that *ame-let-7* would target *Amdop2* [29], and determined that *ame-let-7* abundance decreased with age in honey bees [28]. *Amdop2* expression in the current study showed a reversed trend compared to *ame-let-7*, with an age-related increase. We hypothesized that *Amdop2* could be regulated by miRNA *ame-let-7* in the brain which in turn could regulate worker behaviors. As expected, *ame-let-7* overexpression significantly inhibited *Amdop2* expression in forager brains. Conversely, inhibition of *ame-let-7* significantly increased *Amdop2* expression in foragers. Moreover, luciferase assay confirmed that *ame-let-7* targets the coding region of *Amdop2* because transfection of psiCHECK2-*dop2*-CR reduced the luciferase activity and psiCHECK2-*dop2*-CR mutant rescued this suppression to the same level as that of the blank control. These results strongly indicate that *ame-let-7* directly targets *Amdop2*.

DA receptors mediate gene expression at transcriptional level through its downstream messenger pathways [40]. The mammalian D1 receptor is activated after coupling with multiple transcription factors, such as *zif-268* and *jun-b* at the mRNA level [41], and the cyclic AMP-response element binding protein at the protein level [42]. Guo et al. (2018) confirmed that DA receptors can also act as post-transcriptional regulator [24]. They reported that *Dop1* inhibited *miR-9a* to modulate locust olfactory attraction by inducing the dissociation of La protein. Their results suggest that combined action at two levels by DA receptor is beneficial for regulating gene expression and for controlling rapid behavioral changes. In the current study, overexpression of *ame-let-7* in the forager brains made them more excited, showing stronger sucrose responsiveness compared to the control bees. The observed behavioral phenotype was similar to decreased *Amdop2* mRNA expression, this suggests that *ame-let-7* regulated the transcripts of *Amdop2* to modulate the sugar response of foragers. Taken together, *Amdop2* was mediated by DA [2], and may also be regulated by *ame-let-7*, with lower *Amdop2* causing the stronger sugar response of foragers. Our results further confirmed the function of DA receptors at post-transcriptional level. The sugar responsiveness of honey bee correlates with many behavioral parameters such as age of first foraging, pollen vs nectar/water foraging and learning [27, 43]. We found that *ame-let-7* targeted *Amdop2* to affect foragers’ sugar responsiveness, but it is unclear what behavioral parameters it will impact. Both *ame-let-7* and *Amdop2* have abundant expression in the honey bee brain [29, 44], which is only 1 mm^3^ in size, containing 950,000 neurons, and is accessible to recording and manipulation [45]. Whether *ame-let-7* would affect the neuron function of bees by targeting *Amdop2* should be further explored.

## Conclusion

In summary, feeding of *dsAmdop2* can significantly decrease the expression of *Amdop2* in the brain, which enhanced the sucrose responsiveness of foragers. *ame-let-7* directly targets the coding region of *Amdop2*. Moreover, overexpression of *ame-let-7* enhanced the sucrose responsiveness in foragers, which is similar to the effect of decreased *Amdop2* in foragers. These findings suggest that *ame-let-7* targets *Amdop2* to regulate the sucrose responsiveness of foragers, and may play important roles in regulating honey bee behavior.

### Supplementary Information

**Additional file 1: Table S1.** The sequences of pri-miRNA of ame-let-7, Amdop2-CR-wt and Amdop2-CR-mut.

**Additional file 2: Fig. S1.** A schematic representation of the principle behind the luciferase assay (A). Sequences of the interaction sites between ame-let-7 and Amdop2. Grey shaded areas indicate canonical 7mer “seed” region that aligns with the target site, Asterisks indicate mutated sites, mutated nucleotide bases are shown in bold. The vertical lines indicate contiguous Watson-Crick pairing (B).

## Data Availability

Not applicable.
