# Supplementary material for: MicroRNA ame-let-7 targets Amdop2 to increase sucrose sensitivity in honey bees (Apis mellifera)
Source: Front Zool. 2023 Dec 18;20:41. doi: 10.1186/s12983-023-00519-7 (PMC10726540; doi:10.1186/s12983-023-00519-7)
Supplement: Supplementary file 1 — Additional file 1: Table S1. The sequences of pri-miRNA of ame-let-7, Amdop2-CR-wt and Amdop2-CR-mut. [file 12983_2023_519_MOESM1_ESM.pptx]

## Slide 1
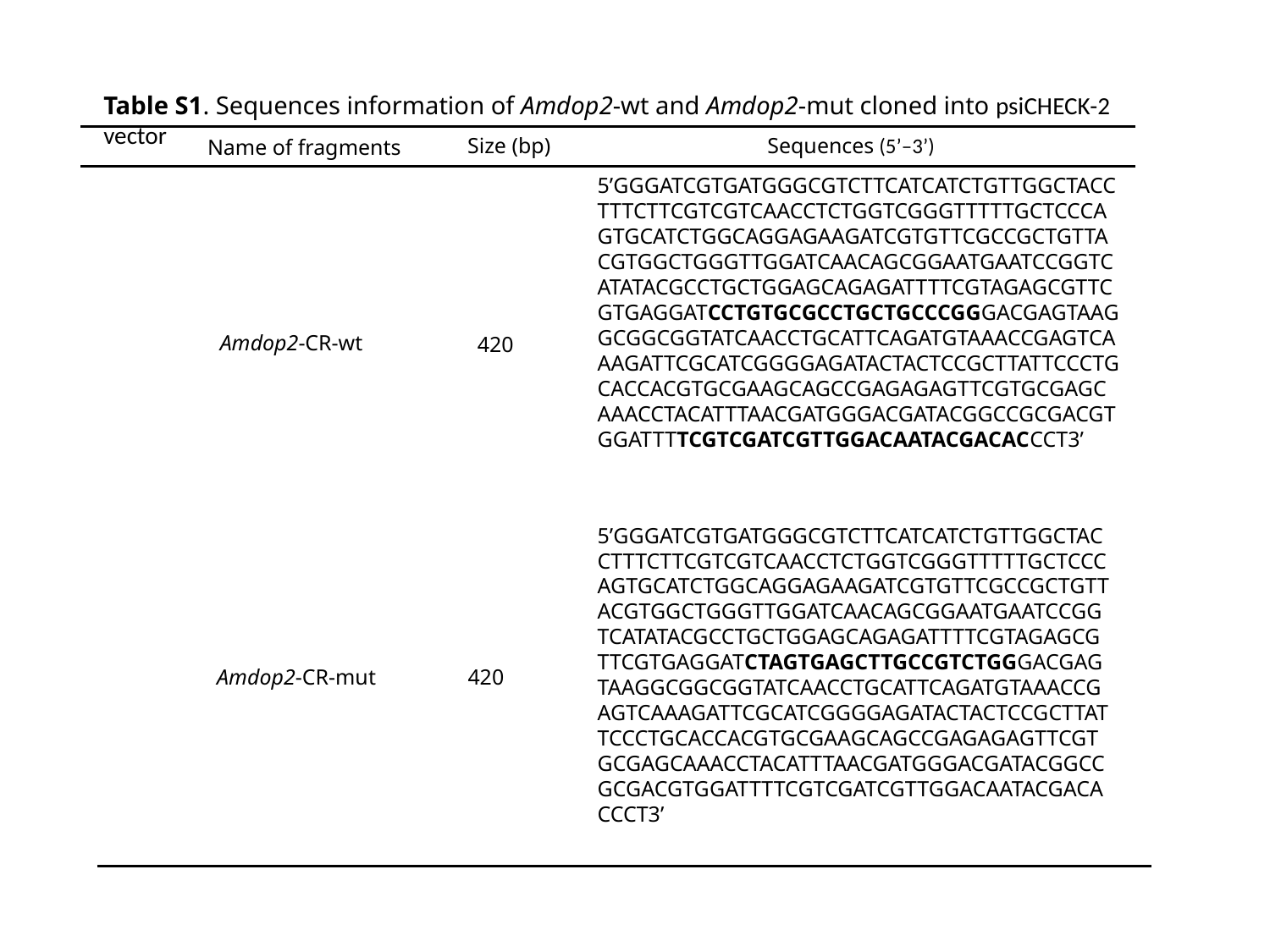

Table S1. Sequences information of Amdop2-wt and Amdop2-mut cloned into psiCHECK-2 vector
5’GGGATCGTGATGGGCGTCTTCATCATCTGTTGGCTACCTTTCTTCGTCGTCAACCTCTGGTCGGGTTTTTGCTCCCAGTGCATCTGGCAGGAGAAGATCGTGTTCGCCGCTGTTACGTGGCTGGGTTGGATCAACAGCGGAATGAATCCGGTCATATACGCCTGCTGGAGCAGAGATTTTCGTAGAGCGTTCGTGAGGATCCTGTGCGCCTGCTGCCCGGGACGAGTAAGGCGGCGGTATCAACCTGCATTCAGATGTAAACCGAGTCAAAGATTCGCATCGGGGAGATACTACTCCGCTTATTCCCTGCACCACGTGCGAAGCAGCCGAGAGAGTTCGTGCGAGCAAACCTACATTTAACGATGGGACGATACGGCCGCGACGTGGATTTTCGTCGATCGTTGGACAATACGACACCCT3’
5’GGGATCGTGATGGGCGTCTTCATCATCTGTTGGCTACCTTTCTTCGTCGTCAACCTCTGGTCGGGTTTTTGCTCCCAGTGCATCTGGCAGGAGAAGATCGTGTTCGCCGCTGTTACGTGGCTGGGTTGGATCAACAGCGGAATGAATCCGGTCATATACGCCTGCTGGAGCAGAGATTTTCGTAGAGCGTTCGTGAGGATCTAGTGAGCTTGCCGTCTGGGACGAGTAAGGCGGCGGTATCAACCTGCATTCAGATGTAAACCGAGTCAAAGATTCGCATCGGGGAGATACTACTCCGCTTATTCCCTGCACCACGTGCGAAGCAGCCGAGAGAGTTCGTGCGAGCAAACCTACATTTAACGATGGGACGATACGGCCGCGACGTGGATTTTCGTCGATCGTTGGACAATACGACACCCT3’
Size (bp)
Sequences (5’–3’)
Name of fragments
Amdop2-CR-wt
420
Amdop2-CR-mut
420
