# Supplementary material for: MicroRNA ame-let-7 targets Amdop2 to increase sucrose sensitivity in honey bees (Apis mellifera)
Source: Front Zool. 2023 Dec 18;20:41. doi: 10.1186/s12983-023-00519-7 (PMC10726540; doi:10.1186/s12983-023-00519-7)
Supplement: Supplementary file 2 — Additional file 2: Fig. S1. A schematic representation of the principle behind the luciferase assay (A). Sequences of the interaction sites between ame-let-7 and Amdop2. Grey shaded areas indicate canonical 7mer “seed” region that aligns with the target site, Asterisks indicate mutated sites, mutated nucleotide bases are shown in bold. The vertical lines indicate contiguous Watson-Crick pairing (B). [file 12983_2023_519_MOESM2_ESM.pptx]

## Slide 1
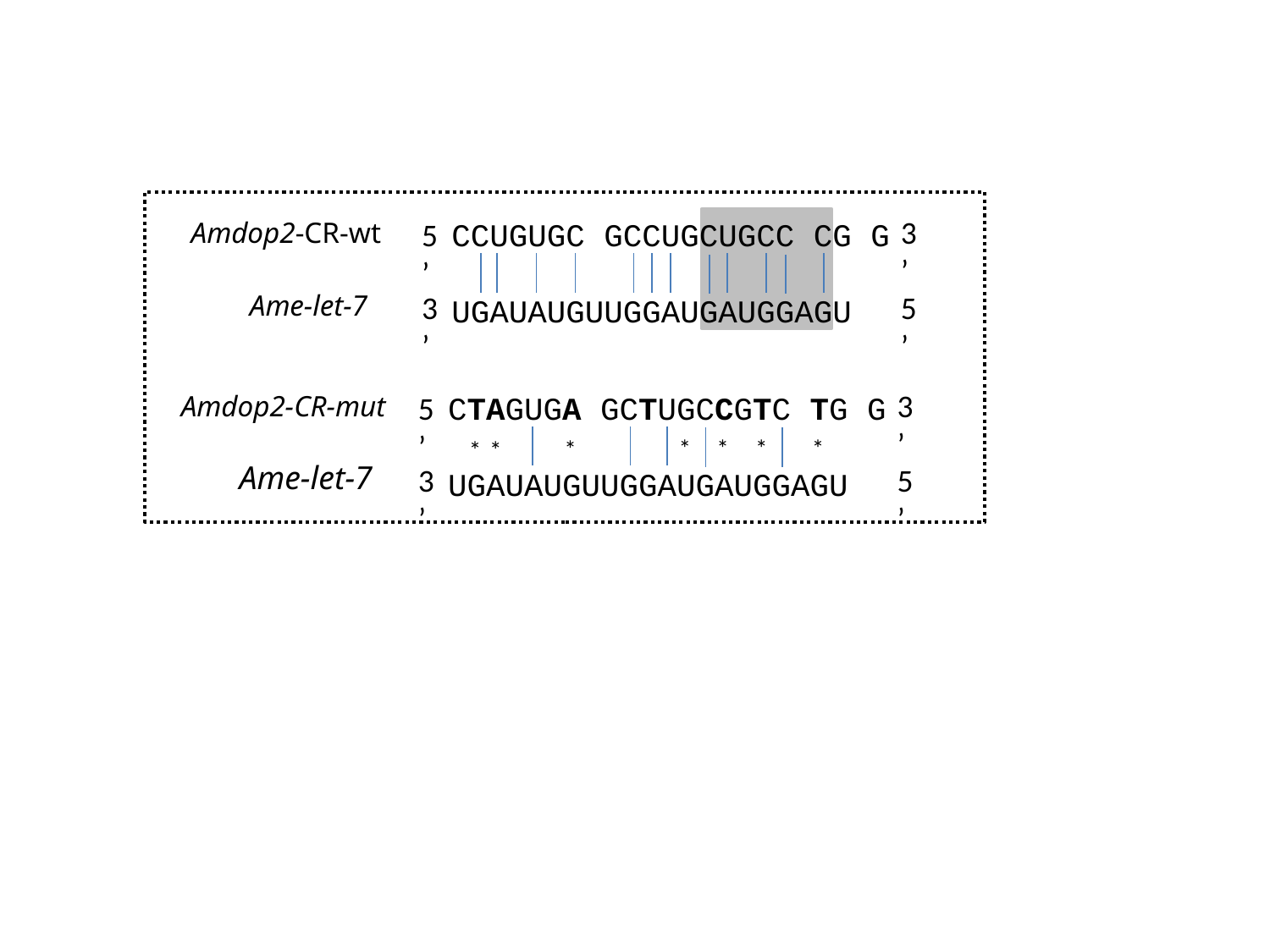

3’
5’
Amdop2-CR-wt
CCUGUGC GCCUGCUGCC CG G
UGAUAUGUUGGAUGAUGGAGU
Ame-let-7
3’
5’
3’
5’
Amdop2-CR-mut
CTAGUGA GCTUGCCGTC TG G
UGAUAUGUUGGAUGAUGGAGU
*
*
*
*
*
*
*
 Ame-let-7
3’
5’
